# Some considerations on digital health validation

**DOI:** 10.1038/s41746-019-0173-2

**Published:** 2019-10-17

**Authors:** M. H. van Velthoven, C . Smith

**Affiliations:** 1 University of Oxford, Oxford, UK; 2 Nagasaki University School of Tropical Medicine and Global Health, Nagasaki, Japan; 3 London School of Hygiene and Tropical Medicine, London, UK

**Keywords:** Health care, Medical research

Mathews et al. propose an ambitious pathway for validation of digital health and mention that a pilot study and detailed corresponding framework are forthcoming.^1^ While we are generally supportive of initiatives to improve the usefulness and value that digital health can offer, we have some considerations and suggestions. Firstly, it needs to be further clarified which types of interventions the digital health scorecard will deal with as a ‘one-size fits all’ approach for validation is unlikely to succeed. As Mathews et al. describe, digital health includes a wide range of scientific concepts, technologies, functions, and users.^1^ For example, if we only consider health apps, not even one initiative will be able to provide a framework for scoring all the 300,000+ and the 200+ mobile health apps, which are added daily cited by the authors.^2^ Most of these apps do not fall under medical device regulation or even have a ‘medical purpose’ but are aimed at improving wellbeing and fitness. The gatekeeper in this aspect are the app stores and, for example, Apple’s App store has created a checklist of items that health apps should adhere to.^3^ Collaborating with app stores is one way to ensure that published health apps at least adhere to basic quality standards.

Secondly, defining the scope of a digital health scorecard will aid in proving its value before considerable amounts of efforts and resources are being spent. There are some similarities between the emergence of the internet as a popular source of health information two decades ago and digital health apps over the past years. The Internet has been a source for medical disinformation and misinformation, which can be argued to have reduced the credibility of science and medicine, similarly for many health apps.^4^ Previously, many called for and developed quality schemes of health information websites, as is now also the case for health apps. There are intrinsic difficulties in scoring the quality of online health information and such schemes need to demonstrate their benefit to the public and patients,^5^ which we think might also the case for health apps and other digital health interventions.^6^ Identifying and quantifying risks is more challenging in the science of benefit-risk assessment as risks depend on the hard to predict consequences of interactions of interventions with other variables in real-life situations. Assessment of the safety of new digital health technologies is not well established and should be undertaken more rigorously. This would help health technology assessment organisations and regulatory bodies to measure the efficacy and safety of new digital health technologies such as apps in robust and reliable ways, while not stifling useful innovation.

Thirdly, as the authors outline in their review paper, ‘usability is arguably among the most important considerations with patient-oriented mobile and digital-based solutions’. Figure 2 outlines the traditional product lifecycle with the proposed digital health scorecard added and starts with ‘needs’ in the developer section. We suggest that input from user and subject experts is crucial to determine these ‘needs’ from the start of the process.^7^

Fourthly, to establish ‘a multi-stakeholder approach that objectively and rigorously evaluates solutions’ as the authors describe, lessons from similar initiatives in other countries should be learned from. Previous multi-stakeholder initiatives to reach consensus on validation of digital health have not reached their initial aims. For example, the European Commission established a working group of different stakeholders to develop mHealth assessment guidelines. After 2 years of work, it came to the conclusion that ‘no consensus was reached among the represented constituencies on a set of guidelines.^8^ This work is based on the guidance from different European countries, including the UK health apps library that is mentioned in the review article, as well as other initiatives such as the French Haute Autorité de Santé (HAS) good practice guidelines. These initiatives differ substantially and providing consensus has proved to be the main challenge.^4^

Fifthly, Mathews et al. acknowledge complexity in this area and that the digital health scorecard would be an approach that evolves over time.^1^ As far as we are aware in most digital health evaluation initiatives no input has been sought from countries outside the OECD. Digital health solutions have considerable value to low- and middle-income countries too as smartphones and other digital tools are increasingly being adopted in these settings. The views of stakeholders in these settings will need to be considered too to provide contextual information that will be crucial for the development and evaluation of appropriate digital health tools.^9^

We think that a digital health scorecard is an ambitious but potentially useful initiative. To optimise its chances of succeeding, it would be helpful to clarify with which digital health interventions it will deal with, in which settings and with which purposes. Different approaches will need to be used to develop and evaluate the increasing number of different digital health interventions. Also, we suggest that before pursuing a pathway for validation, its need and value should be proven because multi-stakeholder approaches that aimed to reach consensus on digital health validation have shown to be challenging, time- and resource-consuming in the past.
